# A neurocomputational model of developmental trajectories of gifted children under a polygenic model: When are gifted children held back by poor environments?

**DOI:** 10.1016/j.intell.2018.06.008

**Published:** 2018

**Authors:** Michael S.C. Thomas

**Affiliations:** a Developmental Neurocognition Lab, Centre for Brain and Cognitive Development, Birkbeck University of London, UK; b Centre for Educational Neuroscience, University of London, UK

**Keywords:** Giftedness, Computational modelling, Artificial neural networks, Cognitive development, Socio-economic status, Behavioural genetics

## Abstract

From the genetic side, giftedness in cognitive development is the result of contribution of many common genetic variants of small effect size, so called polygenicity (Spain et al., 2016). From the environmental side, educationalists have argued for the importance of the environment for sustaining early potential in children, showing that bright poor children are held back in their subsequent development (Feinstein, 2003a). Such correlational data need to be complemented by mechanistic models showing how gifted development results from the respective genetic and environmental influences. A neurocomputational model of cognitive development is presented, using artificial neural networks to simulate the development of a population of children. Variability was produced by many small differences in neurocomputational parameters each influenced by multiple artificial genes, instantiating a polygenic model, and by variations in the level of stimulation from the environment. The simulations captured several key empirical phenomena, including the non-linearity of developmental trajectories, asymmetries in the characteristics of the upper and lower tails of the population distribution, and the potential of poor environments to hold back bright children. At a computational level, ‘gifted’ networks tended to have higher capacity, higher plasticity, less noisy neural processing, a lower impact of regressive events, and a richer environment. However, individual instances presented heterogeneous contributions of these neurocomputational factors, suggesting giftedness has diverse causes.

## Highlights

• Simulates cognitive development in populations of children, with genetic and environmental sources of variation in ability
• Captures gifted development as a polygenic effect – the cumulative effect of many small neurocomputational advantages
• Considers environmental effects as variations in the level of cognitive stimulation
• Causes of variation in ability are quantitative and continuous but bottom and top tails of population behave differently
• Captures role of a rich environment to sustain early gifted development, observed in longitudinal data (Feinstein, 2003a)
• Shows how cases of gifted developmental may be highly heterogeneous at a neurocomputational level

## Introduction

The causes of giftedness in cognitive or physical abilities are complex, involving both genetic and environmental contributions (Sternberg & Davidson, 2005). Humans with exceptional abilities may have innate potential, but skills must be developed over time, and an individual requires a combination of ambition, opportunity and a willingness to work in order to realise their potential; in this sense, Wai (2014) described experts as *born then made*. Moreover, genetic and environmental factors may be correlated. For example, parents may identify an indication of talent in their children and encourage the talent to flourish through providing opportunities and resources (Ericsson, Nandagopal, & Roring, 2005). Talented children may themselves seek out the environments and activities that will foster development of their abilities (Ericsson, 2014).

Recent work in behavioural genetics has focused on genetic contributions to giftedness. Evidence from twin studies in several countries suggested a genetic contribution to cognitive performance in the high range (Haworth et al., 2009). In these data, genetic influences explained 50% of the variance in those performing in the top 15% of population distributions. Molecular genetics using genome wide association (GWA) analyses suggest that the causes of low performance in the bottom tail of the distribution and high performance in the upper tail may be different, at least for intelligence. Spain et al. (2016) found that while the bottom tail was associated with increased incidence of genetic mutations (rare alleles), the upper tail had, if anything, a reduced frequency of rare alleles. The upper tail appears to be driven by the same genetic influences that operate throughout the rest of the population distribution, with the discontinuity at the lower extreme being the sole exception (Shakeshaft et al., 2015). The wider picture is that genetic contributions to intelligence stem from many common genetic variations each of small effect, known as the ‘polygenic’ model (Plomin & Deary, 2015); rare functional variants are more often detrimental than beneficial to intelligence.

Lykken (2006); see also Simonton, 1999, Simonton, 2005 argued that the genetic contributions to giftedness were multiplicative, such that if any of a set of genetic variants was absent, this would negate a gifted outcome – the so-called *emergenic* model. However, twin studies have suggested the genetic contributions to giftedness for intelligence appear to be additive in effect rather than dominant (that is, identical twins are not more than twice as similar as fraternal twins). Plomin and Deary (2015) concluded that twin studies of intelligence consistently find the genetic influence to be largely, if not entirely, additive for high intelligence as well as the entire distribution of intelligence (see also Plomin & Haworth, 2009) – although small or rare non-additive effects cannot be definitely ruled out due to the lack of statistical power to detect them. In sum, then, genetic influence on cognitive ability appears to involve many genes each contributing small effects; these contributions are additive; and for high ability, these genes are common variants. The innately gifted individual has been lucky enough to inherit cognitively beneficial versions of many genes.

Behaviour genetics generates these insights from correlational analyses. However, genetic effects must ultimately unpack in causal properties of the brain and body. With respect to the former, such properties may be construed in terms of neural mechanisms and neurocomputational properties. In these terms, gifted performance is the result of many small advantageous aspects of neurocomputation, potentially across multiple systems, and their contribution to the development and maintenance of cognitive and physical abilities.

A separate literature in educational achievement has focused on environmental influences on the development of children with different levels of ability. Taking a long-term perspective, this literature highlights the role of socio-economic status (SES) in either fostering or holding back early potential. In a seminal paper, Feinstein (2003a) presented an analysis of longitudinal data, grouping children by cognitive ability at 22 months, and then following these children through to 10 years of age. Children from low SES families (where SES was defined by parental education level) did not, on average, ‘overcome the hurdle of lower initial attainment, combined with continued low input’ (Feinstein, 2003b, p. 30). But notably, social inequalities also appeared to dominate the early positive signs of academic ability for most of those low SES children who did well early on. The message that policymakers took from these data was that bright children from poorer families tend to fall back relative to more advantaged peers who have not performed as well (Feinstein, 2015).

**Fig. 1:**
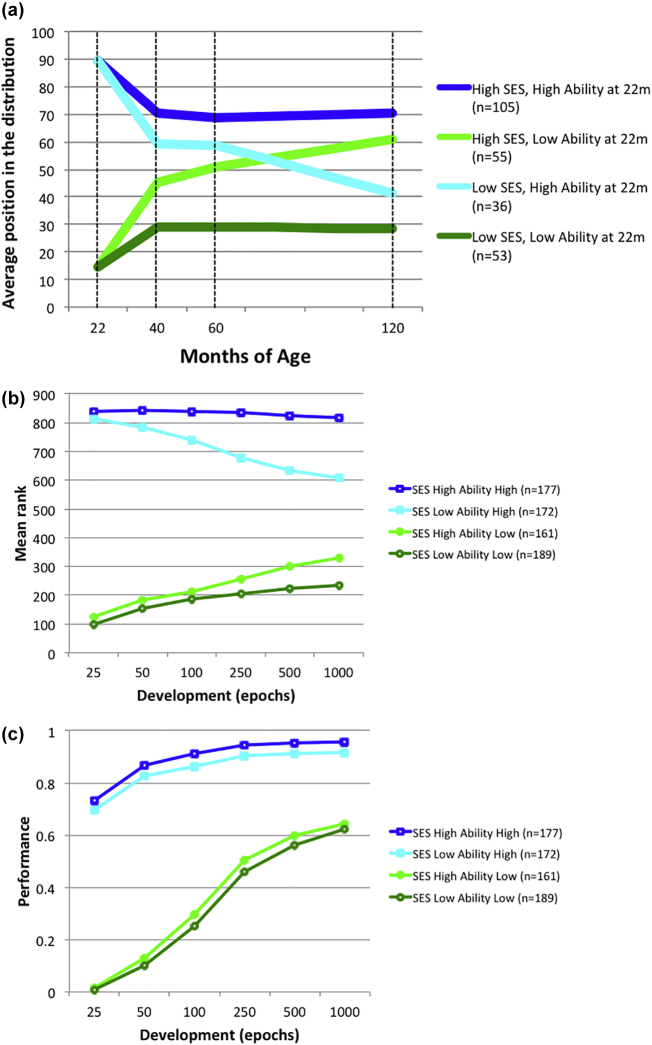
(a) Average rank of children's test scores on cognitive tasks at 22, 42, 60 and 120 months by SES of parents and early rank position (replotted from Feinstein, 2003b). High / Low Q = quartile of cognitive ability assessed at 22 months of age. (b) Simulated data for the population neurocomputational model, with ability assessed at 25 epochs of training, and population rank then measured at 50, 100, 250, 500, and 1000 epochs of training. (c) Simulated developmental trajectories of performance for the four sub-groups.

This pattern is depicted in later Fig. 1(a) replotted from Feinstein (2003b). It shows the population rank order of children classified by ability in the top quartile and bottom quartile on cognitive tests at age 22 months, and then those groups split into high SES (top 24% of population) and low SES (bottom 13%). The top quartile ability / low SES group shows a declining mean rank across age, while the bottom quartile ability / high SES group shows an increasing mean rank. There has been some subsequent debate about the shape of this function: whether the rank trajectories of these two groups really cross, and whether some of the pattern is explained by regression to the mean of initially extreme scores, due to measurement error in the repeated cognitive testing (Jerrim & Vignoles, 2013). However, there is consensus on the main finding: the benefits of good early development can be substantially eroded by social class effects.

Nevertheless, as with data from genetic studies, investigations of environmental influences on the development of children with high ability remain correlational. They stand in need of a mechanistic account that identifies how the proxy of SES translates into actual influences that shape the development of cognitive abilities in children.

In this paper, we use neurocomputational modelling of cognitive development to focus on the mechanistic basis of genetic and environmental influences on high ability. Considering development across a whole population, artificial neural network models are employed to integrate data across levels of description: from the genetic level in terms of influences on neurocomputation; from the environmental level in terms of influences on the level of stimulation children receive from the environment; and from the behavioural level, in terms of scores on cognitive tests.

In previous work, we have shown how modelling cognitive development using populations of artificial neural networks can provide a unified framework to consider individual differences within a developmental framework and integrate across levels of description (Thomas, Forrester, & Ronald, 2016). We have shown that observed SES effects on language development can be simulated by modulating the richness of linguistic experience received by children in families of different SES levels (Thomas, Forrester, & Ronald, 2013). Moreover, this model simulated the asymmetric quality of high and low tails observed in genetic studies: SES predicted whether simulated individuals would fall in the top 10% of the population, but not if they would fall in the bottom 10%. This is because there are many ways to fail but few to succeed: therefore the predictive power of a single factor is reduced for poor outcomes. This novel prediction was subsequently confirmed by a re-analysis of empirical data collected by Bishop (2005). We also investigated the causes of delayed development in this model framework, following the trajectories of simulated children who exhibited early delay (Thomas & Knowland, 2014). Of these individuals, two thirds subsequently resolved to the normal range later in development. This replicates a pattern observed in the empirical literature (e.g., Dale, Price, Bishop, & Plomin, 2003). The model once more produced a novel prediction: that SES should predict variance in the final language ability level of children whose early delay resolved, but not in those where the delay persisted. Once more, this prediction was confirmed by the empirical data (Bishop, 2005).

The modelling framework has therefore demonstrated its initial adequacy to investigate the mechanistic basis of individual differences. In the current work, the Thomas et al. (2013) model is employed to address the developmental trajectories of ‘gifted’ simulated children falling in the upper tail of early performance. Our key questions are as follows: (1) For those simulated individuals showing high early ability, what are the neurocomputational and environmental factors that predict the long-term outcome of developmental trajectories? (2) In a mechanistic model of experience-dependent development, where all sources of variation are specified and there is no measurement error, can the Feinstein graph be replicated, with the population rank order of gifted individuals from lower SES backgrounds subsequently declining across development? (3) If such a decline is observed, must the computational causes of the changes in rank be entirely environmental, as proposed? (4) If changes in population rank are not entirely environmental, can the risk of subsequent decline be predicted from behavioural measures taken when early giftedness is first recognised?

## 1 Computational modelling

### 1.1 Simulation details

#### 1.1.1 Base model

The base model was drawn from the field of language development, and specifically the acquisition of the English past tense within inflectional morphology. The model is used here to stand for more general models of cognitive development utilised in cognitive modelling (see e.g., Mareschal & Thomas, 2007). The model employed an artificial neural network architecture.

**Fig. 2:**
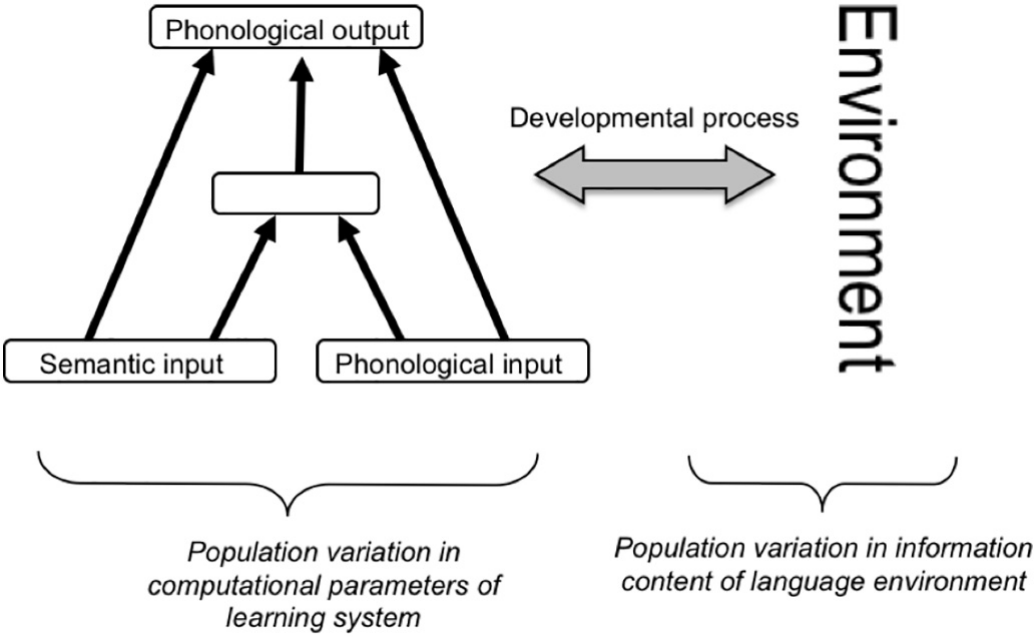
Schematic of the population simulations (from Thomas et al., 2013).

A backpropagation network was used to learn to output the past-tense form of a verb from an input vector that combined a phonological representation of the verb stem and lexical-semantic information (Joanisse & Seidenberg, 1999). The architecture is shown in Fig. 2.

The training set was the “phone” vocabulary from Plunkett and Marchman (1991). This comprised an artificial language set constructed to reflect many of the important structural features of English past-tense formation. There were 500 monosyllabic verbs, constructed using consonant-vowel templates and the phoneme set of English. Phonemes were represented over 19 binary articulatory features (e.g., voicing, tongue position, lip shape), a distributed encoding based on standard linguistic categorisations (Fromkin & Rodman, 1988). Separate banks of units were used to represent the initial, middle, and final phonemes of each monosyllable. The output layer incorporated an additional 5 features to represent the affix for regular verbs. The input layer included 500 units to encode the lexical status of each verb in the training set using a localist encoding scheme (Joanisse & Seidenberg, 1999; Thomas & Karmiloff-Smith, 2003). Networks thus had 3 × 19 + 500 = 557 input units and 3 × 19 + 5 = 62 output units. The connectivity, including number of internal units, between input and output layers could vary (see below).

**Fig. 3:**
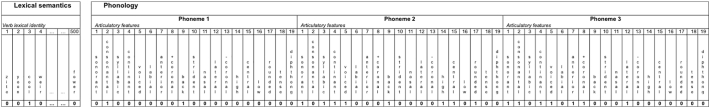
Coding scheme to encode each verb as a distributed pattern of activation over a set of units in the input of the artificial neural network. The training set comprised 500 artificial verbs, constructed using the phonology of English. A small number of consonant-vowel templates were used to build tri-phonemic verb stems. In addition to phonological input, lexical semantic information represented the identity of each verb. The output of the network was the phonological past tense form of each verb. The training set was built to capture the key structure of the English past tense system (Plunkett & Marchman, 1991). The coding scheme shows the lexical semantic input and phonological feature set for the verb ‘cool’ (/k/ /U/ /l/), with the regular past tense ‘cooled’.

There were four types of verbs in the artificial language training set: (1) regular verbs that formed their past tense by adding one of the three allomorphs of the + rule, conditioned by the final phoneme of the verb stem (e.g., analogously for English, *tame-tamed*, *wrap-wrapped*, *chat-chatted*); (2) irregular verbs whose past-tense form was identical to the verb stem (e.g., *hit-hit*); (3) irregular verbs that formed their past tenses by changing an internal vowel (e.g., *hide-hid*); (4) irregular verbs whose past-tense form bore no relation to its verb stem (e.g., *go-went*). There were 410 regular verbs, and 20, 68, and 2, respectively, of each irregular verb type. A separate set of novel verbs was constructed to evaluate the generalisation performance of the network. These verbs could differ depending on their similarity to items in the training set. Generalisation in this case was assessed via 410 novel verbs each of which shared two phonemes with one of the regular verbs in the training set, and was evaluated based on the proportion of these novel verbs that were assigned the correct allomorph of the regular past-tense rule. The coding scheme for a sample artificial verb is shown in Fig. 3.

#### 1.1.2 Encoding extrinsic variation

Each network simulated a child raised in a given family, and families were assumed to vary in the richness of the language used. The language input was assumed to vary to some extent according to SES (Hart & Risley, 1995). A training set was created for the past-tense information available in each family environment. SES was implemented through generating a *family quotient* for each simulated child. The family quotient was a number between 0 and 100%. This value was used as a probability determining whether each verb in the full training set would be included in the family's vocabulary. The family training set was then fixed throughout development. Performance was always assessed against the full training set (analogous to a standardised test of past-tense formation applied to all children). The family quotient manipulation corresponded to a reduction in type frequency for both regular and irregular verbs. Based on the findings of Thomas et al. (2013) on the appropriate range of intrinsic versus extrinsic variation to capture data on past tense acquisition, family quotients were sampled from a uniform distribution from 60% to 100%, producing learning environments of reasonably high quality. This corresponds to the assumption that there is a minimum amount of linguistic information typically available to a child. In the analyses, a family quotient value of 80% was used to split simulated individuals into high (>80%) and low (<80%) SES groups.

#### 1.1.3 Encoding intrinsic variation

Artificial neural networks contain a range of parameters that increase or decrease their ability to learn a given training set. Parameters such as learning rate, momentum, and number of hidden units feature in most published simulations. In models of normal/average development, parameters are optimised to achieve best learning (usually in the presence of the full training set). In the current model, a number of parameters were simultaneously varied across individual networks, with learning ability determined by their cumulative effect. Thomas, Forrester, and Ronald (2016) showed how these parameters could be encoded in an artificial genome under a polygenic coding scheme, and Thomas (2016) showed how twin study designs could be simulated by creating networks whose artificial genomes were identical (for identical twins) or shared 50% of genes on average (for fraternal twins). Importantly, the population distribution in learning ability was simulated by the accumulation of many small influences from different neurocomputational parameters, themselves influenced by multiple genes. Individual gene-behaviour correlations were therefore of very small effect (Thomas, Forrester, & Ronald, 2016).

Variations occurred over sixteen neurocomputational parameters, allowing for over 2000 billion unique individuals. The parameters were as follows: *Network construction*: Architecture, number of hidden units, range for initial connection weight randomisation, and sparseness of initial connectivity between layers. *Network activation*: unit threshold function, processing noise, and response accuracy threshold. *Network adaptation*: backpropagation error metric used in the learning algorithm, learning rate, and momentum. As well as an overall learning rate, there were separate parameters modifying the learning rate between the semantic input units and the hidden units, and the phonological input units and the hidden units, potentially altering the relative balance of these sources of information during learning, and therefore allowing more lexical or phonological strategies to past-tense acquisition. *Network maintenance*: weight decay, pruning onset, pruning probability, and pruning threshold.^1^

In previous artificial neural network models of cognitive development, most of these parameters have been varied separately to account for individual differences or disorders, pursuing hypotheses that, for instance, differences in behaviour may arise from differences in underlying neural plasticity or from the actions of certain neurotransmitters. These models have addressed the development of abilities such as language, reading, category formation, reasoning and selective attention. For example, variations in architecture have been used to explain dyslexia: Zorzi, Houghton, and Butterworth (1998); hidden units to explain intelligence Richardson, Baughman, Forrester, & Thomas, 2006, Richardson, Forrester, Baughman, & Thomas, 2006 and autism Cohen, 1998; sparseness of connectivity to explain autism: McClelland (2000); processing noise to explain Specific Language Impairment: Joanisse and Seidenberg (2003); unit threshold function to explain schizophrenia Cohen & Servan-Schreiber, 1992 and aging Li & Lindenberger, 1999; connection pruning to explain autism: Thomas, Knowland, and Karmiloff-Smith (2011); and learning rate to explain general intelligence: Garlick (2002). For the current model, the hypothesis was that within a population, differences between individuals in the quality of neurocomputation arise from simultaneous small variations in many low-level parameters. Following the central limit theorem, the combination of a large number of independent variables will tend toward a normal distribution.

Two of the parameters had categorical values: architecture (direct connections between input and output layer, indirect connections via an intermediate hidden layer, a combination of both) and learning algorithm error metric (root mean squared error or cross-entropy in backpropagation). The other parameters had continuous values. Their range was calibrated in the following way: An initial ‘normal’ set of parameters was defined. These were estimated based on previous research. Each of the continuously valued parameters was then varied in turn, holding the all other parameters at their initial values. For each parameter, the range was derived that produced failure of learning up to highly successful learning. In some cases, parameters had a monotonic relationship to performance (e.g., hidden units, where more was better); in other cases, there was an optimal intermediate value (e.g., activation function). The aim was to determine an average or adequate value for each parameter, which was defined heuristically as ‘just enough to succeed and then a little bit more’. Values were then derived that would cause increasingly poorer or increasingly better performance around this value. An attempt was made to make poorer and better performance roughly symmetrical around average performance for each parameter. The parameter ranges were as follows: hidden units (6 to 500), temperature of logistic function specifying unit threshold (0.0625 to 4), noise added to net input (0 to 5), general learning rate (0.005 to 0.5), learning rate modifier from semantic input units (0 to 1); learning rate modifier from phonological input units (0 to 1), momentum (0 to 0.75), weight variance (0.005 to 2.25), response threshold (0.005 to 0.5), pruning onset epoch (0 to 1000), pruning probability (0 to 1), pruning threshold (0.1 to 1.5), weight decay (0 to 984.3 × 10^−7^), sparseness (0 to 0.7).

Lastly, an artificial genome for each individual specified their values across this parameter set. Artificial genes were binary, and set randomly to 0 or 1 to create a population. Sets of genes were allocated together to determine the value of each neurocomputational parameter, the number of genes depending on how many values each parameter could take in the population. The artificial genome had a total of 156 bits: hidden units: 12; temperature: 12; noise: 10; learning rate: 12; phonological learning rate: 12; semantic learning rate: 12; momentum: 8; weight variance: 10; architecture: 8; learning algorithm: 4; nearest neighbour threshold: 6; pruning onset epoch: 10; pruning probability: 8; pruning threshold: 10; weight decay: 12; sparseness: 10. For a given parameter, the value was determined by adding the number of 1 s (e.g., how many 1 s out of 12 for hidden units) and using a lookup table to derive the parameter value. The binomial distribution ensured that intermediate values of each parameter were more common in the population, and extreme high or low values less common (all look-up tables are available via the link in footnote 1). Artificial genes independently influenced each parameter, implementing polygenicity but no pleitropy.

In sum, the normal distribution of learning ability in the population arose from two sources, the large number of neurocomputational parameters being combined in each individual, and the tendency for these values to more often have intermediate than extreme values.

#### 1.1.4 Processing roles

Despite the large number of parameters, these can be viewed as serving a smaller number of processing roles within the network (although some parameters contribute to more than one role). Some parameters alter the network's learning *capacity*, that is, the complexity and the amount of information that can be learned. These include the architecture, the number of hidden units, and the initial sparseness of connectivity. *Regressive events* involving pruning of connections can also reduce capacity later in development, implicating the pruning onset, pruning probability and pruning threshold parameters in predicting learning trajectories (see Thomas et al., 2011). The nature of the learning algorithm determines both what can be learned and also how quickly. The speed of learning can be thought of as the network's *plasticity*. Other parameters alter plasticity, including the learning rate parameter, the learning rates in semantic and phonological connections, the momentum, the initial range of weight variation, and the unit threshold function. The unit threshold function determines how responsive a processing unit is to variations in its input, and therefore to some extent determines the quality of the *signal* propagating through the network. Signal is also affected by the level of processing noise, and the accuracy required of output units to drive a behavioural response. Combined with the quality of the learning environment, the mechanisms affecting development can be broadly assigned the following four categories: *capacity*, *plasticity*, *signal*, and *regressive events*. Parameters are categorised in this way in the reporting of results.

#### 1.1.5 Design

Development was traced across a population of 1000 simulated individuals, focusing on the rate of acquisition of regular English past-tense forms. One thousand sets of the 16 computational parameter values were generated via randomly created genomes, with gene alleles sampled independently. Corresponding computational parameter sets were instantiated as 1000 artificial neural networks. A family quotient value was generated for each network and used to create an individualised family training set. Each network was trained for 1000 epochs on its family training set, where one epoch constituted a presentation of all the items in the individual network's family training set. At each epoch, performance was measured on the full training set. Performance was assessed on regular verbs, irregular verbs, and on generalisation of the past-tense rule to novel forms, in order to generate a behavioural ‘profile’ for each network. Performance was measured via accuracy levels (% correct).

## 2 Results

### 2.1 Stability of ability level over development

One question considered by Feinstein (2003a) was whether later educational attainment, say at 10 years of age, could be predicted by early measures of a child's cognitive ability, say around the age of 2. Such prediction is compromised by several factors, including measurement error, the need to use different tasks at different ages and, even where a common task is used, the possibility that the child will perform the same task using different cognitive processes at different ages. These issues aside, Feinstein presented evidence of some stability in cognitive test scores across age. Performance on cognitive tasks at 22 months of age correlated with those at 10 years of age at around 0.2. At 42 months, scores correlated with age-10 performance at around 0.3. At 60 months, the 10-age correlation was around 0.4. (The stability of cognitive ability tends to dependent, to some extent, on the ages compared, and whether correlations are computed between individual ability scores or latent variables. E.g., Sameroff, Seifer, Baldwin, & Baldwin, 1993, reported correlations of 0.72 between age 4 and 13; Trzaskowski, Yang, Visscher, & Plomin, 2014, demonstrated a correlation between the genetic component of cognitive ability, inferred through a twin study, of 0.75 between age 7 and 12).

For the simulation, we assessed performance of the set of networks across development, at 25, 50, 100, 250, 500 and 1000 epochs on regular verbs, where 1000 epochs was the end of training. Regular verbs formed the largest proportion of the training set. We use 250 epochs notionally to correspond to middle childhood. Performance at the earliest stage, 25 epochs, correlated with the 250-epoch measure at 0.65. Performance at 50 epochs correlated with the 250-epoch measure at 0.80, and that at 100 epochs at 0.91. The correlation between the early measure and performance at the end of training (notionally, adulthood) was 0.52. All correlations are shown in Table 1.

**Table 1:** Developmental stability of individual differences in the ability of simulated children.

Epoch	50	100	250	500	1000
25	0.920⁎	0.776⁎	0.651⁎	0.570⁎	0.524⁎
50		0.913⁎	0.804⁎	0.715⁎	0.659⁎
100			0.912⁎	0.841⁎	0.789⁎
250				0.974⁎	0.942⁎
500					0.985⁎

Data show Pearson correlations between performance on regular verbs at different time points in development, for six points: 25, 50, 100, 250, 500 and 1000 epochs of training. (N = 1000 simulated children).

⁎ Pearson correlation, p < 0.000001.

In the model, there was no measurement error, and the same task was assessed across development. Correlations in the model were therefore unsurprisingly higher than the empirical data. As with the empirical data, performance in middle childhood was better predicted by earlier measures that were closer in time. The more the initial measure preceded middle childhood, the weaker the predictive power. In these non-linear artificial neural networks, therefore, there is some stability in the relative performance of networks as they acquire the learning domain. However, trajectories were non-linear and their relative position also changed to some extent. Even in the controlled framework of the simulations, performance at the earliest time point only explained 27.4% of the variance in the final level of performance across the population.

### 2.2 The Feinstein analysis

At the same early point in development, 25 epochs, a population rank order of performance on regular verbs was established as a measure of each network's ability, with a rank of 1000 as the best and 1 as the worst. High ability networks were defined as having a rank above 650, and low ability networks were defined as having a rank below 350. The family quotient, an index of the quality of the environment, served as the implementation of SES. It varied between 60% and 100%. A value of 80% was used to split simulated individuals into high (>80%) and low (<80%) SES groups. The combination of high versus low ability at 25 epochs, and high versus low SES, generated four groups. (Note, the rank cut-off values of 650 and 350 were chosen to generate good numbers of networks in the four subgroups, with all n > 150. Since the training environment already affected network performance by epoch 25, more extreme cut-offs tended to produce few networks in the high-ability/low-SES and the low-ability/high-SES groups).

Per the method of Feinstein (2003a), the mean rank order for each of the four subgroups was calculated at 25 epochs, and then at five subsequent stages in development, at 50, 100, 250, 500, and 1000 epochs of training. Fig. 1(b) shows the results, alongside Feinstein's original data (Feinstein, 2003b). The simulation results demonstrate relative rank stability in the high-ability/high-SES and low-ability/low-SES groups. As with the empirical data, initially high-ability/low-SES networks subsequently showed a decline in mean rank order, while those from initially low-ability/high-SES groups showed an increasing mean rank. Because measurement error was absent in the simulation, these data demonstrate that the convergence of these two groups in mean rank is not solely the result of regression to the mean. While the convergence of the two groups is not as steep as in Feinstein's data, the degree of convergence in the empirical case has been argued to depend on the cut-offs used to define initial high and low ability groups (Washbrook & Lee, 2015). In both Washbrook and Lee's empirical analysis and in the simulated data, less extreme definitions of high and low ability tended to produce steeper convergence; see Thomas (2018) for a more detailed analysis of this effect. Finally, plots of rank order can exaggerate the size of the actual differences in performance level. Fig. 1(c) demonstrates the mean accuracy level of the four sub-groups across development, showing relatively subtle divergences between trajectories.

In sum, the population simulation was able to qualitatively capture the empirical pattern reported by Feinstein (2003a), whereby bright children from poorer families tend to fall back relative to their more advantaged peers; and it did so by implementing SES as a variation in the richness of the structured learning environment to which children are exposed.

### 2.3 Predictors of individual trajectories of early gifted individuals

**Fig. 4:**
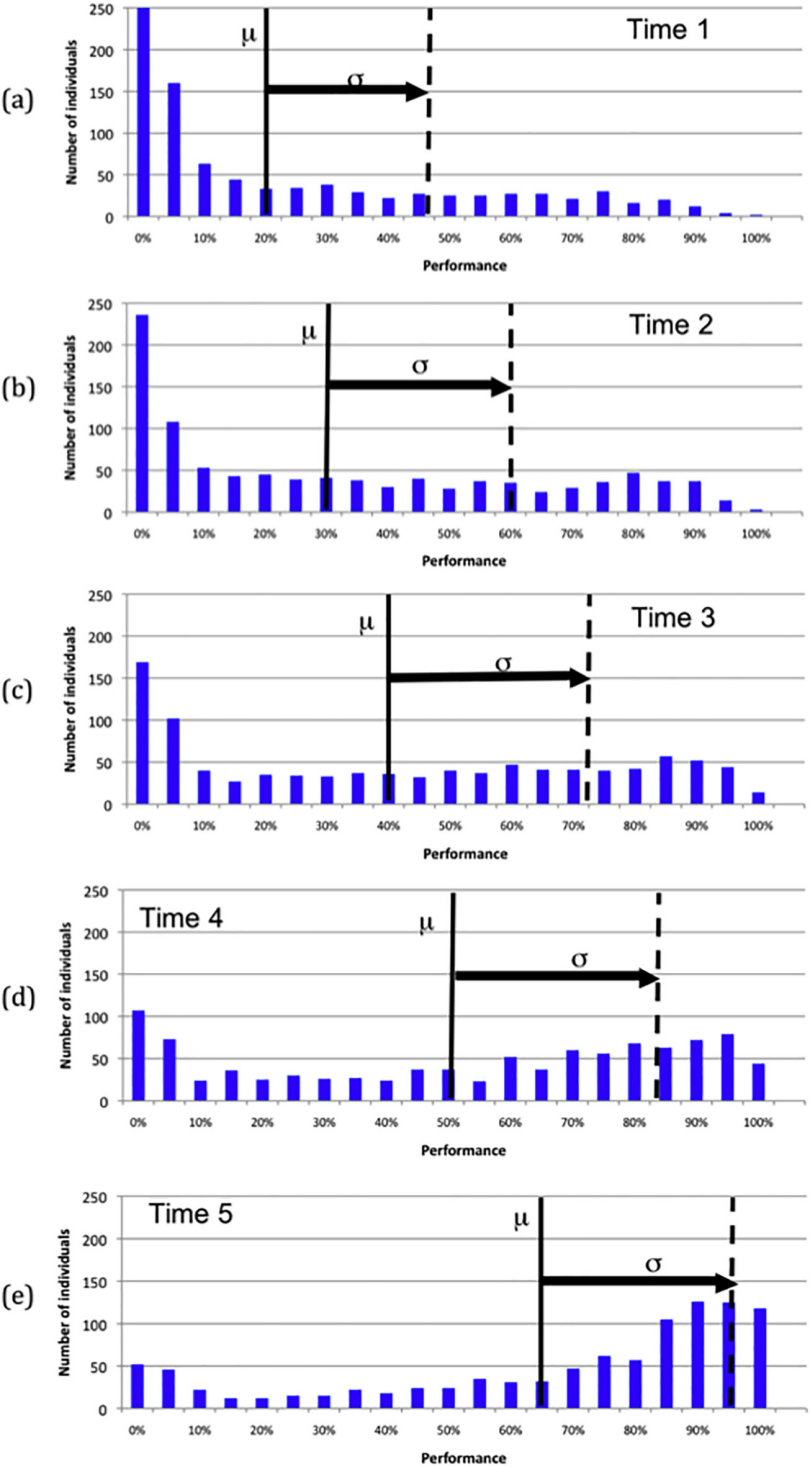
Performance distribution on regular verbs at each time point, along with the cut-off for defining giftedness. μ is the mean and σ is the standard deviation at each time point.

In order to track the development of individuals with early potential, five time points were identified, based on mean population accuracy levels on regular verbs. These were when the population accuracy was 20, 30, 40, 50 and 65%, occurring respectively at 13, 21, 31, 49, and 127 epochs of training. The earlier time 1 point gave the best opportunity to spot the fast developers, while the time 5 point was chosen because after this point, population mean plus 1 standard deviation exceeded 100% accuracy – with ceiling performance possible, the rest of the population can catch up. At each of the five time points, gifted individuals were defined as those falling >1 standard deviation (σ) above the population mean (μ). Fig. 4 shows the distribution of population performance at each time point, along with the cut-off (μ + 1 * σ) to define the gifted group.

At the first time point, the group of gifted individuals corresponded to 20.3% of the population, comprising 44 from the lowest SES quartile, 35 and 58 from the middle quartiles, and 66 from the upper quartile. The highest two SES quartiles had the most individuals, and this pattern differed reliably from chance (X(3) = 11.40, p = 0.010). The richness of the training environment was therefore instrumental in producing gifted performance even at this early point in training. However, 44 (21.7%) of early diagnosed gifted came from individuals exposed to the poorest environments (bottom quartile).

Trajectories were followed to assess whether individuals initially identified as gifted retained this status at each measurement point, or whether they returned to the normal range. We refer to this henceforth at ‘renorming’. While 44 of early diagnosed gifted came from individuals exposed to the poorest environments, all but one of these individuals subsequently renormed. The initial implication is that without the support of a rich environment, gifted performance will not sustain.

**Fig. 5:**
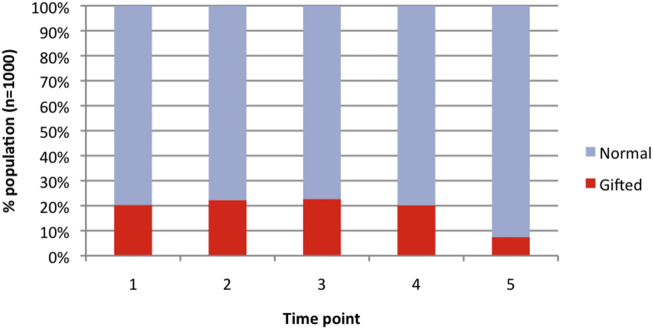
Proportion of simulated population exhibiting giftedness at each time point, where giftedness was defined as falling >1 standard deviation above the population mean at that time point (see Fig. 4).

Fig. 5 depicts the proportion of the population classified as gifted at each time point. The proportion dropped over developmental time. Of those initially classified, gifted individuals renormed in 63.5% of cases. Sustained giftedness was observed in only 36.5% of individuals (7.4% of the population). This reduction predominantly occurred at the final time point, and was partly due to the top of the normal range approached ceiling performance on the regular verb measure, making it harder for individuals to fall above the normal range. It was expected that at time 5, any early gifted individuals who had renormed would nevertheless remain toward the top of the normal range. Of those renorming, 94.4% (134 individuals) indeed remained in the top 500 of the population.

**Fig. 6:**
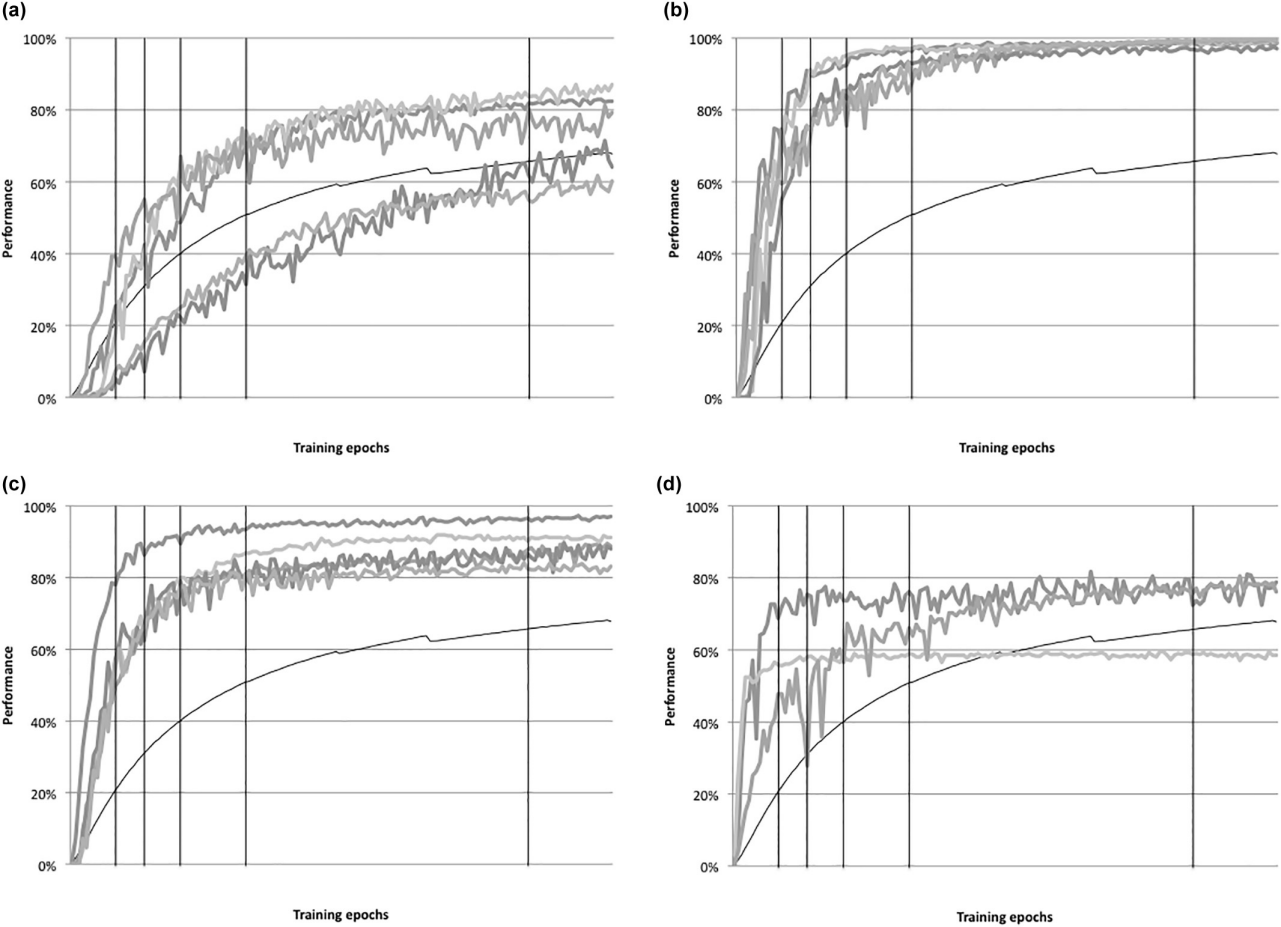
Sample developmental trajectories for regular verbs, for each group: (a) non-gifted development, (b) sustained gifted, (c) renorming gifted with high outcome, (d) renorming gifted with low outcome (excluding instances of overt regression). Trajectories are shown for the first 150 epochs of training, to delineate the earliest phases of development. The final time point to assess outcome was 127 epochs. The black line represents the mean trajectory for the entire population. (Dinks in this line represent epochs were pruning was activated in different individuals, causing dips in performance in a few vulnerable individuals.)

Only in a handful of cases was final performance poorer after early strong development: in 4.3% (6 individuals), final performance was in the bottom 500, and in 1.4% (2 individuals), final performance was in the bottom 250. Of those finishing with poor outcomes, 5 out of the 8 individuals demonstrated developmental regression, where the trajectory showed an overt drop in performance at some point in training, followed by recovery. In these cases, a chance combination of risk factors led the connection-pruning process to cause damage to established connectivity and therefore performance to decline following strong early development. Elsewhere we have considered this process as a candidate mechanism for developmental regression in autism (Thomas et al., 2011; Thomas, Davis, Karmiloff-Smith, Knowland, & Charman, 2016), and we do not consider these cases further here. Trajectories for the three remaining individuals with strong early development but low final outcome are shown in Fig. 6(d), and the reasons for these profiles are considered as case studies below as a demonstration of the causal heterogeneity of gifted profiles.

We saw in the introduction that genetic data have pointed to a causal asymmetry between the lower and upper tails of the distribution of cognitive development (Spain et al., 2016). Using the Thomas et al. (2013) model, Thomas and Knowland (2014) followed the trajectories of simulated individuals exhibiting early signs of delayed development (>1 standard deviation below the population mean at time 1). For these individuals, the early delay resolved in around two thirds of cases. For the cases of resolving delay, approximately 20% of cases led to good eventual outcomes (in the top half of the population). For cases of giftedness, however, very few individuals who returned to the normal range then demonstrated a low (bottom half) final rank order in the population (5.7%, or 2.1% excluding cases of overt regression). Although the model produced a distribution of development by continuous quantitative variations across a range of parameters, it demonstrated asymmetries in the respective tails of the distributions, where early delay was sometimes associated with good final outcome, but early advantage was not typically associated with poor final outcome. Wedded with the observation that, in this model, environmental quality predicts whether individuals will fall in the top tail but not the bottom tail (Thomas et al., 2013), the model demonstrates asymmetric properties in development at the respective extremes from continuous underlying causal factors.

Fig. 6 depicts sample trajectories from each of four groups: non-gifted, sustained gifted, renorming gifted with high final performance (top 500), and the few cases of renorming gifted with low final performance (bottom 500). The figure also indicates the mean for the whole population, and the five measurement time points. It depicts early development across the first 150 epochs of training (the fifth time point used to assess outcome was at 127 epochs). Our principal interest in the remaining analyses is to understand the specific neurocomputational parameters responsible for each type of developmental profile within the model, and whether different types of trajectories could be predicted from behavioural profiles at the earliest time point. The popular view of the Feinstein data – and the one that attracted the attention of policymakers – is that the environment drives changes in rank order across development in gifted individuals.

### 2.4 Predicting gifted trajectories from neurocomputational parameters and SES

We first investigated which parameters, intrinsic or extrinsic, predicted non-gifted, sustained gifted, or renorming outcomes at the final time point. Different options were available for performing this analysis: by assessing whether there were reliable between-group differences for each parameter; via a multinomial logistic regression with the parameters as predictors and the group category as the outcome; or via a linear discriminant analysis to identify which linear combination of parameters best discriminated each pair of groups. Each method has limitations, including possible violations of assumptions of the ANOVAs, in terms of normal distribution of scores and unequal groups, as well as multiple comparisons; limitations in the fit of the logistic regression model to the data, and use of linear combinations of parameters when computationally, artificial neural network parameters are primarily non-linear in the way they interact. Both ANOVA and logistic regression methods are presented here and are compared in Table 2. Linear discriminant analyses were also run and produced similar results. The table shows pair-wise group comparisons, including effect sizes and statistical significance for those parameters where one or other reliably distinguished the groups. Parameters are grouped by their processing roles identified in the Methods.

**Table 2:** Neurocomputational parameters that reliably discriminated between groups.

Parameter	Role	N-G vs. S-G		N-G vs. RN		S-G vs. RN	
		ANV	MLR	ANV	MLR	ANV	MLR
Hidden units	Capacity	⁎⁎**0.036**	⁎⁎32.1	⁎⁎**0.024**	⁎6.5		
Architecture	Capacity	⁎⁎**0.008**					
Sparseness	Capacity			⁎⁎**0.009**	⁎⁎13.7		
Pruning onset	Capacity	⁎**0.006**				⁎⁎**0.038**	
Pruning prob.	Capacity						
Pruning threshold	Capacity	⁎**0.005**					
Learning algorithm	Capacity / Plasticity	⁎**0.007**					
Learning rate (l-r)	Plasticity	⁎⁎**0.025**	⁎5.6	⁎⁎**0.043**	⁎⁎8.3		
Semantic l-r	Plasticity	⁎⁎**0.022**	⁎4.7			⁎⁎**0.063**	⁎6.6
Phonological l-r	Plasticity	⁎⁎**0.021**		⁎⁎**0.024**	⁎3.8		
Momentum	Plasticity	⁎**0.005**		⁎⁎**0.008**			
Weight variance	Plasticity	⁎⁎**0.015**		⁎⁎**0.015**	⁎4.3		
Temperature	Plasticity/Signal		⁎⁎13.7				
Noise	Signal	⁎⁎**0.008**		⁎⁎**0.015**			
NN-threshold	Signal	⁎⁎**0.029**	⁎⁎7.2	⁎⁎**0.059**	⁎⁎14.5		
Weight decay	Signal						
Family quotient (SES)	Environment	⁎⁎**0.080**	⁎⁎16.4			⁎⁎**0.292**	⁎⁎27.9

MLR model fit: S-G vs. RN, X(18) = 98.4, p < 0.001, Nagelkerke R^2^ = 0.557.

MLR model fit: N-G vs. S-G, X(18) = 117.4, p < 0.001, Nagelkerke R^2^ = 0.319.

MLR model fit: N-G vs. RN, X(18) = 211.1, p < 0.001, Nagelkerke R^2^ = 0.361.

N-G = not gifted. S-G = sustained gifted. RN = renorming gifted (early gifted but later returning to the normal range). Results are shown for two complementary statistical analyses. ANV = analysis of variance (shown in **bold**); scores show partial eta-squared effect sizes. MRL = multinomial logistic regression; scores show Wald statistic for each parameter.

Empty cells represent non-reliable differences (p > 0.05).

⁎ effect reliable at p < 0.05.

⁎⁎ effect reliable at p < 0.01.

In line with the polygenic model of giftedness, several parameters distinguished sustained gifted from non-gifted individuals, with hidden unit number and family quotient (SES) explaining most of the variance. Gifted networks typically had higher capacity, higher plasticity, processing less disrupted by noise, a lower impact of regressive events, and a richer environment. The role of environment in gifted outcomes contrasts with its lack of power in predicting delay observed by Thomas and Knowland (2014). The renorming gifted group differed from non-gifted via similar parameters to the sustained gifted, but with generally smaller effects. Sustained gifted reliably differed from renorming gifted only in a few parameters. The renorming gifted group had, respectively, earlier pruning onset, lower semantic learning rate, and a poorer environment. The renorming group had values on these parameters similar to the non-gifted group, while sustained gifted had different values. (Appendix Table A shows the mean parameter values for the three groups).

To be early gifted implies the ideal combination of a range of parameters, both intrinsic and extrinsic. The parameters distinguishing sustained and renorming gifted may be understood as follows. Sustained gifted had later pruning onset and so could use their connectivity resources for longer (indeed, via longer growth, these connections were perhaps immunised to loss because they had the opportunity to grow stronger). Sustained gifted had a higher semantic learning rate. This enables this particular type of network to adopt an effective task learning strategy: to use semantic input to facilitate exception verb learning and so allow the phonological pathway to learn regular verbs. This delivered a better computational division of labour for processing this particular domain. The sustained gifted networks had a richer environment, whereas the renorming had a normal environment, on average.

The overall picture at the level of mechanism is that sustained gifted performance required a combination of stronger computational properties and enriched environment. In line with the Feinstein analysis, early promise could be lost without aid from a rich environment. Notably, however, in the model, neurocomputational parameters *also* contributed to changes in rank order across development.

### 2.5 Using early behavioural profiles to predict gifted outcomes

Taking a perspective blind to the neurocomputational properties of each network, profiles of behavioural scores were assessed at time 1 to see whether those with outcomes of sustained giftedness could be distinguished from those with renorming giftedness. Could developmental outcomes be predicted from early behavioural markers, blind to processing properties? The behavioural profile was initially constructed from a rich combination of domain-specific measures tapping performance on aspects of English past tense morphology, including accuracy of production of both regular and irregular verbs, and generalisation of inflectional patterns to novel forms with similarity either to regulars or irregulars, measuring generalisation of the rule and for the latter also measuring generalisation of the irregular pattern (see van der Lely & Ullman, 2001; Thomas et al., 2001, for the use of such a profile with typical and atypical developing children). However, the results can be simplified to three of these measures, performance on regulars, irregulars, and rule generalisation to novel verbs that rhymed with regular verbs in the training set. The means for these three measures are shown in Table 3.

**Table 3:** Time 1 (13 epochs) mean (standard deviation) performance per group (% correct for Regular and Vowel-change Irregular Verbs, % regularized for Novel regular rhymes), for giftedness groups defined by early regular verb performance.

Group	Regular	Exception	Novel
Not gifted (797)	8.69 (12.84)	1.00 (2.75)	7.40 (11.18)
Sustained gifted (61)	70.51 (13.39)	16.08 (12.39)	56.04 (13.36)
Renorm high outcome (134)	65.91 (11.84)	11.55 (11.70)	54.75 (11.52)
Renorm low outcome (6)	59.43 (9.99)	13.97 (19.70)	47.48 (9.03)
Renorm poor outcome (2)	48.29 (0.34)	16.18 (22.88)	36.95 (1.21)

The groups' early performance differed reliably on regular verbs (F(1,193) = 5.82, p < 0.001, n = 0.029) and on all types of exception patterns (no-change: F(1,193) = 16.92, p < 0.001, n = 0.081; vowel-change: F(1,193) = 6.07, p = 0.015, n = 0.030; arbitrary: F(1,193) = 7.24, p = 0.008, n = 0.036); but did not reliably differ on early generalisation performance to novel rhymes (F(1,193) = 0.477, p = 0.491, n = 0.002). That is, sustained giftedness could be distinguished from renorming giftedness at the point where the groups were defined, based on performance on the *training set*, but not generalisation performance. Sustained gifted individuals acquired knowledge more quickly but did not differ in extracting the structure of the domain to generalise to new inputs. Again, this result contrasts with early behavioural markers that predicted the outcome of delay (Thomas & Knowland, 2014). For delay, persisting versus resolving delay could be predicted based on early differences in extracting regular structure from the training set, that is, on regular verb performance and on generalisation performance, while there was no difference on exception performance. In sum, in the model, the outcome of early giftedness was predicted behaviourally by the speed of acquiring knowledge (generalisation being uniformly strong among the early gifted), whereas outcome of early delay was predicted by speed of extracting generalizable structure (the acquisition of irregular verbs being uniformly weak among early delayed).

### 2.6 Neurocomputational heterogeneity in gifted profiles

Under a polygenic model, multiple factors combine to produce giftedness, and the combination of factors may differ across individuals. As an illustration of this point, we examined three individuals classified as early gifted who were subsequently rated in the bottom 500 at time 5 (where 1000 was best and 1 was worst, they had population ranks of 437, 317, and 475, respectively). Appendix Table B contains the full parameter sets for these individual networks, and compares them to the population means for non-gifted, sustained gifted, and renorming gifted individuals. What sets these three individuals apart? The parameters would have to explain first why the individual should show fast early development, and second, why performance should fail to sustain this increase so that population rank declined. In each case, *no single parameter was responsible*; rather several parameters interacted. The trajectories were generated by the interplay between the five effects of capacity, signal quality, plasticity, regressive events, and the environment. Fast early development arose through high plasticity and good signal, while poor final performance was associated with regressive events, limited capacity, or poor environment. Notably, in these three case studies, environment either: (1) played no role in outcome, (2) contributed to early fast development, or (3) contributed to low final performance (see Appendix Table B). Its influence was therefore variable. These case studies are important in demonstrating that *under a polygenic model, single causes of (even unusual) developmental trajectories may be hard to identify*.

## 3 Discussion

Giftedness in children is a complex phenomenon, requiring many circumstances to come together to produce potential, combined with the motivation to work hard to deliver the potential, and an environment to support that development. The genetic contribution to giftedness appears to be polygenic, with common variations in many genes contributing small influences (Spain et al., 2016). It has been argued that the requirement for many beneficial circumstances to align points to a multiplicative model of influences on giftedness, where the absence of any one would scupper the gifted outcome (Lykken, 2006). However, twin studies have pointed merely to additive effects for high cognitive performance (Plomin & Deary, 2015). From the environmental side, Feinstein (2003a) influentially argued that differences in SES could limit the cognitive and educational achievements of bright children, with bright children from low SES families dropping behind those from high SES families.

In this article, we have argued that correlational data need to be complemented by mechanistic accounts, which seek to explain how genetic and environmental influences contribute to the trajectories of cognitive development exhibited by gifted children. Models of cognitive development capturing populations of children offer one way to advance such accounts. Here, we used a neurocomputational model drawn from the field of language acquisition. Population variability in development was produced by many small differences in neurocomputational parameters each influenced by multiple genes, implementing polygenic effects, and by the richness of the stimulation available from the environment, taken to be associated with differences in SES (Hart & Risley, 1995).

The model demonstrated several key findings. First, while there was some stability in simulated children's relative ability levels across development, there were also changes. For instance, some individuals showing early promise later dropped back into the normal range. Performance early in development only predicted 27% of the variance in the ‘adult’ models, even under ideal conditions of zero measurement error, due to the non-linearity of developmental trajectories. This captures the limited predictive power of children's early cognitive skills observed in empirical data (Feinstein, 2003a).

Second, the model replicated the effects of SES on children with different early ability levels reported by Feinstein (2003a). Notably, simulated individuals scoring highly early in development were more likely to fall back in population rank when they were in families with lower SES. Feinstein's graph has proved influential (and controversial) with policymakers. Here, the computational model demonstrated that the pattern can be replicated if SES is assumed to operate by influencing children's levels of cognitive stimulation, and is not an artefact of regression to the mean. Thomas (2018) provides a deeper consideration of factors influencing the shape of this function, including whether it may also arise if SES impacts directly on neurocomputational parameters, and implications for interventions to close developmental gaps between children.

Third, examination of the neurocomputational parameters that produced trajectories of gifted development yielded several findings. An early classification of giftedness required a number of beneficial properties to coincide, including both intrinsic neurocomputational parameters, and a high quality training environment. There were some cases of an early gifted classification without the support from a strong environment, but in almost all cases, these individuals returned to the normal range later in development. In other words, early promise was lost without the subsequent support of a strong environment. A number of factors influenced whether early promise was sustained. Broadly construed, these involved the processing roles of capacity, signal quality, plasticity, regressive events, and environmental richness. Importantly, to the extent that variation in these neurocomputational properties is influenced by genes, children's rank orders in the population may alter across development for genetic reasons. This contrasts with the usual interpretation of the data presented by Feinstein, 2003a, Feinstein, 2003b that changes in population rank solely reflect environmental influences. Lastly, there was some indication that early behavioural profiles could predict whether an individual would exhibit a sustained gifted outcome without knowledge of internal neurocomputational properties. Gifted development in the model was characterised by a fast rate of acquiring knowledge, rather than extracting the regularities of learning domains. However, in line with the non-linearity of developmental trajectories, effect sizes were not large.

Fourth, even with a continuum of mechanistic cause of variation in the population, apparent asymmetries were observed between the influences on gifted versus delayed performance, that is, the tails of the normal distribution. Poor environment was not predictive of delay but good environment was predictive of giftedness (Thomas et al., 2013). Individuals whose early delay resolved could show final levels of performance in the upper half of the population, but early gifted individuals who fell back into the normal range rarely end up in the lower half of the population. And unusual trajectories could be produced that were not explainable by a single cause, but only in terms of the interactions of multiple factors. These characteristics might lead one to conclude that performance in the tails requires special mechanisms or, as in Lykken's emergenic account Lykken (2006), that giftedness can be scuppered by the absence of any of multiple factors, such as not having a rich environment. Nevertheless, in the model, these patterns arose from additive influences on non-linear computational systems.

There are, of course, many limitations to the model. It is a cognitive model (as is required to make contact with behaviour), which restricts the neural plausibility of its design. In other models, these parameters have been linked with neural properties such as brain size, neural plasticity, and neurotransmitter levels (Cohen & Servan-Schreiber, 1992; Garlick, 2002; Thomas, 2016). However, these links are neither precise nor direct. Moreover, although a population was simulated, this is a far cry from simulating a given human population, where a vast range of empirical data must be brought together to constrain the actual genotypes and environments involved and their ranges of variation. Only a single system was modelled, so the target behaviour was relatively simple. The model did not tackle complexities such as the child's motivation, or gene-environment correlations such as where parents offer more stimulation to children who show early talent. Moreover, the implementation of SES presented here does not address the possibility that SES might affect brain development and function, rather than just levels of cognitive stimulation (Hackman, Farah, & Meaney, 2010; see Thomas, 2018).

When we scale up to think about more complex human abilities, these are likely the product of multiple interacting brain systems. For example, research in numeracy skills has used brain imaging methods to reveal the involvement and interactions between systems for the perception of number symbols (in fusiform gyrus in occipto-temporal lobes), for representations of numerosity and manipulation of quantities (respectively, in intraparietal sulcus and the angular gyrus), for spatial abilities (in the parietal lobe), for mathematical language (inferior frontal gyrus), and for concepts, principles and procedures (involving pre-frontal cortex) (Butterworth & Varma, 2013). These systems may be differentially impacted by influences related to SES and by genetic influences on neurocomputational parameters. For example, spatial skills are correlated with mathematical ability in childhood (Gilligan, Flouri, & Farran, 2017), yet these skills appear less influenced by variation in SES than language development (Farah et al., 2006). In contrast to this specificity, behavioural genetic evidence assessing performance across different academic subjects in examinations for 16 year olds suggested both similar heritability of around 50% and also largely shared genetic influence across subjects, from English to mathematics to science, humanities, second language, art and business (Krapohl et al., 2014; Rimfeld, Kovas, Dale, & Plomin, 2015). Such shared genetic influence across diverse high-level academic abilities could indicate that they recruit overlapping, domain-general mechanisms, or that the suite of domain-specific mechanisms involved in each is subject to common genetic influence in their neurocomputational parameters. That is, in a given individual, all domain-specific mechanisms might have similar, superior neuroplasticity or similar low levels of neural noise. To progress this question requires the construction of more complex, multi-component developmental models, which incorporate variation both in neurocomputational properties and in the stimulation these components receive.

Nevertheless, the model of giftedness presented here is a demonstration of the type of mechanistic account that is necessary to explain correlational data at multiple levels of description and to generate novel predictions. A simple associative system, exposed to a psychologically constrained learning environment, was sufficient to capture a range of empirical data regarding gifted development, given certain assumptions about the sources of intrinsic and extrinsic variability. Its simplifications notwithstanding, the current model highlights the importance of rich cognitive stimulation for sustaining the strong cognitive development of children who show early gifted potential.

## Figures and Tables

**Table A:** Mean values for neurocomputational parameters and environmental quality for the non-gifted group (N = 797), the sustained gifted group (N = 61), and the re-norming group (N = 134).

Parameter	Role	Not gifted N = 797	Sustained gifted N = 61	*Re*-norming N = 134
Hidden units	Capacity	28	39	35
Architecture	Capacity	1.00	1.16	1.14
Sparseness	Capacity	0.07	0.05	0.04
Pruning onset	Capacity	104	125	96
Pruning prob.	Capacity	0.13	0.14	0.15
Pruning threshold	Capacity	0.53	0.49	0.53
Learning algorithm	Capacity/Plasticity	0.91	1.00	0.96
Learning rate (l-r)	Plasticity	0.12	0.15	0.15
Semantic l-r	Plasticity	0.53	0.72	0.55
Phonological l-r	Plasticity	0.32	0.49	0.45
Momentum	Plasticity	0.25	0.29	0.28
Weight variance	Plasticity	0.58	0.39	0.44
Temperature	Plasticity/Signal	1.23	1.25	1.31
Noise	Signal	0.67	0.46	0.46
NN-threshold	Signal	0.07	0.12	0.12
Weight decay	Signal	6.7 × 10^−7^	5.4 × 10^−7^	3.5 × 10^−7^
Fam. Quot. (SES)	Environment	0.79	0.92	0.78

**Table B:** Parameter sets for the three case studies that were initially classified as gifted, at outcome were rated as poor (bottom 50% of population), but which did not show overt developmental regression.

Parameter	Role	Case1	Case2	Case2	NG	SG	RN
Hidden units	Capacity	25	25	25	28	39	35
Architecture	Capacity	**0**	**0**	1	1	1.16	1.14
Sparseness	Capacity	0.2	0	0.1	0.07	0.05	0.04
Pruning onset	Capacity	**50**	**0**	**75**	104	125	96
Pruning prob.	Capacity	0.025	**0.5**	0.05	0.13	0.14	0.15
Pruning threshold	Capacity	0.5	**1**	0.5	0.53	0.49	0.53
Learning algorithm	Capacity/Plasticity	1	1	1	0.91	1	0.96
Learning rate (l-r)	Plasticity	**0.15**	**0.175**	**0.175**	0.12	0.15	0.15
Semantic l-r	Plasticity	0.1	0.5	0.1	0.53	0.72	0.55
Phonological l-r	Plasticity	**0.75**	0.5	**0.75**	0.32	0.49	0.45
Momentum	Plasticity	**0.5**	0.1	0.35	0.25	0.29	0.28
Weight variance	Plasticity	0.5	0.5	0.75	0.58	0.39	0.44
Temperature	Plasticity/Signal	0.5	1.25	1.25	1.23	1.25	1.31
Noise	Signal	**0.5**	**0.25**	**0.25**	0.67	0.46	0.46
NN-threshold	Signal	**0.1**	**0.2**	**0.2**	0.07	0.12	0.12
Weight decay ×10^−7^	Signal	1.00	2.00	0	6.74	5.38	3.47
Fam. Quot. (SES)	Environment	0.774	**0.907**	**0.623**	0.790	0.920	0.780

These are compared with the mean values for non-gifted individuals (NG), sustained gifted (SG), and gifted re-norming individuals who remained in the top 50% of the population (RN). Bolded values are those that mark the cases as different from the three groups.

## Appendix

Parameters distinguishing the unusual cases of early giftedness followed by poor outcome were as follows:

• Case 1 had several parameter settings that contributed to fast early development. These included the fast learning rate, phonological learning rate, and momentum, and well as a 2-layer network architecture which changed its weights quickly and was optimal for learning regular verbs (this individual would likely not show early giftedness measured on irregular verbs). In addition, the network had good signal properties, with lower noise and a higher nearest-neighbour response threshold than the non-gifted group (a high NN-threshold meant the network output did not have to be so precise to generate a correct response). Two principal parameter settings appeared to cause the low final outcome: the 2-layer architecture, which had limited capacity; and an early onset of pruning, which cut away resources and reduced capacity further. *Environment appeared to play no role in this case*.
• Case 2 showed strong early development through a combination of high plasticity (a high learning rate), good signal (low noise, high NN-threshold), and *a rich environment*. The decline in performance appeared to be due to regressive events, with pruning in operation from the beginning of training, leading to a steady loss of resources. In addition, the architecture was again 2-layer, implicating a limited capacity. Here, *environment played a role in early giftedness.*
• Case 3 had high plasticity (learning rate) and good signal (low noise, high NN-threshold), but again experienced earlier onset of pruning, and particularly in this case, appeared *limited by a poor environment*. In contrast, the capacity of the network was higher. Here, *environment contributed to the poorer final outcome*.

## Footnotes

1 Detailed specification of the parameters can be found in a technical report available at http://www.psyc.bbk.ac.uk/research/DNL/techreport/Thomas_paramtables_TR2011-2.pdf
